# Myotonia Congenita in Australian Merino Sheep with a Missense Variant in *CLCN1*

**DOI:** 10.3390/ani14243703

**Published:** 2024-12-22

**Authors:** Leah K. Manning, Katie L. M. Eager, Cali E. Willet, Shaun Slattery, Justine H. McNally, Zoe B. Spiers, Mark Hazelton, Georgina Child, Rick Duggan, Brendon A. O’Rourke, Imke Tammen

**Affiliations:** 1 Sydney School of Veterinary Science, The University of Sydney, Camden, NSW 2570, Australia; 2 Elizabeth Macarthur Agricultural Institute, Department of Primary Industries and Regional Development, Woodbridge Road, Menangle, NSW 2568, Australia; 3 Sydney Informatics Hub, Core Research Facilities, The University of Sydney, Camperdown, NSW 2050, Australia; 4 North West Local Land Services, Narrabri, NSW 2390, Australia; 5 North West Local Land Services, Moree, NSW 2400, Australia; 6 Small Animal Specialist Hospital, North Ryde, NSW 2113, Australia

**Keywords:** myotonia congenita, inherited, ovine, whole genome sequencing, *CLCN1*

## Abstract

Myotonia congenita is a hereditary, non-dystrophic skeletal muscle disorder associated with muscle stiffness due to delayed muscle relaxation after contraction. We review myotonia congenita in domesticated animals and humans and investigated suspected myotonia congenita in a flock of Merino sheep in Australia. In 2020, a property in New South Wales reported a four-year history of lambs that would fall on disturbance before rapidly recovering, with 13 affected sheep identified in 2020. Episodes were associated with a short period of tetanic spasms and a stiff gait upon rising. Lambs were otherwise normal between episodes, although over time, lost body condition and occasionally died from misadventure. An inherited condition was considered from limited pedigree information and a preliminary diagnosis of myotonia congenita was made based on clinical presentation. Biochemistry from four sheep found variable, but typically mild increases in creatine kinase (CK) and aspartate aminotransferase (AST). Modified electromyography on six affected sheep found irregular electrical activity within the muscle. For four sheep, there were no consistent significant abnormalities on post mortem examination and histopathology—typical for this condition. A review of the Online Mendelian Inheritance in Man (OMIM) and Online Mendelian Inheritance in Animals (OMIA) databases was conducted to summarise information about myotonia congenita in humans and eight non-human species of animals. Comparing the characteristic clinical presentation, pathology and electromyography data of affected Merino sheep to similar conditions in other species assisted the identification of likely candidate genes. Whole genome sequencing of two affected lambs detected a missense variant in *CLCN1* (NC_056057.1:g.107930611C>T; XM_004008136.5:c.844C>T; XP_004008185.4:p.(P282S)), with a predicted deleterious effect on protein function. An SNP genotyping assay was developed, and the variant segregated with the disease in 12 affected sheep and obligate carrier rams under an assumed recessive mode of inheritance. Identifying a likely causal variant and developing a diagnostic test allows screening of suspected affected or carrier Merino sheep for early intervention to reduce propagation of the variant within flocks.

## Simple Summary

Myotonia congenita is an inherited disease in humans and animals caused by genetic variants in the *CLCN1* gene. The disease is characterised by an inability of muscles to quickly relax after contraction, which can result in stiff movements and frequent falls. We summarise current knowledge about myotonia congenita in domesticated animals and report the disease for the first time in Merino sheep. Affected sheep presented with episodic collapse. Clinical examination, biochemistry, measurement of electrical activity in the muscle, and pathology resulted in a likely diagnosis of myotonia congenita. DNA analysis identified a likely causal variant in the *CLCN1* gene. Determination of this variant has led to the development of a diagnostic test that allows farmers to make informed breeding decisions and reduce the spread of the causal variant within the Australian Merino population.

## 1. Introduction

Myotonia is the condition in which there is sustained contraction of muscle and delayed relaxation following voluntary movement and is detected clinically and on electromyography [1,2,3]. Myotonia may be hereditary or acquired, and hereditary myotonia may occur in dystrophic or non-dystrophic forms [4,5]. Non-dystrophic hereditary myopathies may occur due to ion channelopathies, including abnormalities in chloride or sodium ion channels [1,4,5,6]. A second group of channelopathies, termed periodic paralyses, are associated with calcium, sodium or potassium channel aberrations and present as episodic muscle weakness [6].

Myotonia congenita is a non-dystrophic hereditary myopathy associated with reduced chloride conduction in skeletal muscle due to variants in the chloride voltage-gated channel 1 (*CLCN1*) [3,4]. Abnormal ion conduction increases the electrical excitability of skeletal muscle which manifests as prolonged muscle contraction and delayed relaxation [1,3,4,7,8,9].

Myotonia congenita has been reported in humans [4], mice [10,11,12] and eight non-laboratory animal species (OMIA:000698), with likely causal variants identified in the *CLCN1* gene in all species except cattle for which a single affected calf was reported and a likely causative variant not determined [13,14,15,16,17,18,19,20,21,22,23,24,25]. Myotonia congenita has been reported in humans as both autosomal recessive (Becker disease) and autosomal dominant (Thomsen disease), with Becker disease often more severe [3,4,5,26]. Affected goats, commonly known as fainting goats, have an autosomal dominant form of myotonia congenita, while the other animal species in which causative variants have been recognised have autosomal recessive forms of myotonia congenita [27].

Humans and animals affected by myotonia congenita may have episodes of muscle stiffness that often follow initiation of movement or a stimulus with improvement of signs after exercise [1,4,5]. In animals, this usually manifests as intermittent gait abnormalities and often, but not always, episodic collapse or recumbency [1,5]. Concurrent clinical signs vary but typically relate to episodic abnormal muscle function, such as altered vocalisation, cyanosis, stridor and dysphagia [1,2,18,24]. Localised or generalised muscle hypertrophy is often reported [1,4].

A diagnosis of myotonia congenita in animals is based on clinical presentation including clinical signs and examination (e.g., percussion myotonia), presence of myotonic discharges on electromyography and where available, confirmation of a causative variant by genetic testing [28]. Histopathology of muscle is relatively non-specific, but it can assist in excluding other myopathies.

Myotonia may also be seen in conditions other than myotonia congenita. In humans, myotonia may be seen with conditions such as paramyotonia congenita, potassium-aggravated myotonia, myotonic dystrophy types 1 and 2, Pompe’s disease, other myopathies, or may be drug-induced [3,6]. Acquired myotonia also occurs in animals, such as in canine hyperadrenocorticism, intoxications with drugs that block the chloride channel or drugs that inhibit cholesterol synthesis, and some muscular dystrophies or unclassified myopathies [1,28].

In sheep, myotonia congenita has previously been reported as an autosomal recessive condition in Rasa Aragonesa sheep in Spain, as well as sheep of unspecified breed in a single flock in the USA [15,29]. A missense variant in the second exon of *CLCN1* was implicated as a causative variant in Rasa Aragonesa sheep [15], while a causative variant was not determined in the USA study [29]. This manuscript summarises information about myotonia congenita in humans, companion animals and livestock and describes myotonia congenita for the first time in Merino sheep, associated with a novel variant in the *CLCN1* gene.

## 2. Materials and Methods

### 2.1. Characterisation of the Disease Phenotype

#### 2.1.1. History and Clinical Examination

In 2020, an Australian sheep producer described a four-year history of a syndrome with lambs falling on disturbance before rapidly recovering. The property was a Merino spring-lambing operation with approximately 1700 ewes and 31 rams. Sporadically affected lambs were observed between 2017 and 2019, and 13 affected animals were reported in 2020. Affected lambs were initially identified at marking (approximately six weeks of age) and there was no sex predilection. For lambs identified as affected in previous years, the owner reported they would lose body condition compared to their peers and occasionally die of misadventure. At least one affected lamb had previously survived to two years of age. Property visits and physical examinations of 13 owner-reported affected animals were performed in 2020. Thirteen eight-week-old Merino lambs were yarded and examined in 2020. The remaining affected lambs were assessed again at approximately 5 months of age when electromyography was conducted (Supplementary Table S1).

#### 2.1.2. Clinical Pathology

Blood was collected from four affected lambs at approximately five months of age and complete biochemistry and haematology was performed at Regional Laboratory Services (RLS), Benalla, Victoria, Australia.

#### 2.1.3. Electromyography

Electromyography was performed under field conditions on six affected lambs (Supplementary Table S1) and three unaffected age-matched lambs at approximately five months of age. Lambs were placed in lateral recumbency and an electromyography was conducted on the quadriceps, triceps, gastrocnemius and extensor carpi radialis muscles using AdInstruments Powerlab26 T + bioamp cable (Bella Vista, Australia), normal needle electrodes and Natus ultra disposable monopolar needle electrode (Newington, Australia), using a 50 × 0.45 mm 26-gauge needle and 0.75 lead wire. Data were collected and examined using Lab Chart 8.1.22, AdInstruments (Bella Vista, Australia). Any voluntary movement was documented in association with electromyography readings.

#### 2.1.4. Gross Pathology and Histopathology

Four affected 10.5-week-old lambs were humanely euthanised with pentobarbitone intravenous injection due to long-term welfare concerns in a commercial setting (Supplementary Table S1). A complete post mortem examination was performed on four lambs. Tissue samples were fixed in 10% formalin, processed and embedded in paraffin wax and 4 µm sections were cut and stained with haematoxylin and eosin (H&E). Sections of brain examined included obex, cerebellum, mesencephalon, thalamus and cerebral cortex. Muscle sections examined included tongue, biceps brachii, diaphragm, gastrocnemius, gluteal and paravertebral muscles. Selected sections were stained with Luxol Fast Blue (LFB) with a Periodic Acid-Schiff counterstain.

#### 2.1.5. Parentage Verification

DNA extraction was performed on RNA*later* (ThermoFisher, Waltham, MA, USA) stabilised spleen tissue for four affected animals and on EDTA blood for nine additional affected animals and 31 unaffected rams using the DNeasy blood and tissue kit (Qiagen, Germantown, MD, USA) following the manufacturers protocol. Samples were quantified using the Qubit instrument and HS assay kit (ThermoFisher). Collection of samples in this study was approved by the University of Sydney Animal Ethics Committee (Project number 2020/1720). Parentage verification was performed on an Ion Torrent S5XL sequencer (ThermoFisher) using a modified Ampliseq protocol incorporating over 400 SNPs recommended for parentage analysis by the Australian Sheep CRC. A half-volume reaction protocol was used to amplify the custom SNP panel with 10 ng of DNA and the Ion AmpliSeq Library kit V2 (ThermoFisher). Sequencing alignment and QC was performed using the Torrent Suite software v5.12.0 and plugins CoverageAnalysis v5.12.0.0 and VariantCaller v5.12.0.2. Hotspot data were exported and parentage analysis was performed by Cervus v3.0.7 [30] to qualify or exclude candidate sires. Parentage relationships were qualified where there was 0–1 mismatch between sire and offspring.

### 2.2. Candidate Genes

A shortlist of candidate genes was created by searching for similar phenotypes on Online Mendelian Inheritance in Man (OMIM) [31] and Online Mendelian Inheritance in Animals (OMIA) [27]. A search on OMIM was conducted to include “myotonia AND channelopathy”. An advanced search in OMIA was performed by extracting phenes with the categories ‘Nervous system phene’ and ‘Muscle phene’. Genes associated with disorders that had similar history and clinical findings to the Merino sheep, such as collapse with or without muscle rigidity, episodic clinical signs and an absence of overt neurologic abnormalities such as tremors and seizures were selected.

### 2.3. Identification and Validation of a Likely Causal Variant in the Functional Candidate Gene CLCN1

#### 2.3.1. Genome Wide Association Study (GWAS)

DNA from six affected animals identified in 2020 (Supplementary Table S1) was sent for high density SNP chip genotyping using the Illumina Ovine 600K BeadChip by an external service provider (Neogen, Lansing, MI, USA). GWAS were performed using PLINK v1.90b6.21 [32,33] and visualised using the qqman package in RStudio, R version 4.2.3v0.1.9 [34]. A basic case/control association test was performed using the 6 affected animals and 144 control sheep, which were genotyped as part of other in-house research projects (Merino *n* = 89, other breeds *n* = 55).

#### 2.3.2. Whole Genome Sequencing (WGS) Analysis

DNA from two affected animals was sent for whole genome sequencing by an external service provider (Azenta, Suzhou, Jiangsu Province, China). WGS data from 23 unrelated animals (Merino *n* = 17, other breeds *n* = 6) generated as part of other in-house research projects were used for joint analysis. Data quality for all samples was visually assessed using FastQC v. 0.11.7 [35]. Samples that showed adapter sequence greater than 5% were trimmed using the bbduk program in BBTools v39.01 (bbduk.sh: https://sourceforge.net/projects/bbmap/, accessed on 6 October 2022). Read pairs were aligned to the ARS-UI_Ramb_v2.0 reference genome (GCF_016772045.1, accessed on 17 October 2023) using DRAGMAP v1.3.0 (https://github.com/Illumina/DRAGMAP, accessed on 16 August 2023). Samples that had multiple BAMs were merged to one BAM per sample with SAMbamba v. 0.8.1 [36]. Sorting and indexing were performed with SAMtools v 1.1 [37] and duplicate reads were marked with SAMBLASTER v 0.1.24 [38].

Short variants (SNPs and indels) were processed following the Sydney Informatics Hub Germline-ShortV pipeline [39]. Samples were called with GATK HaplotypeCaller v. 4.4.0.0 [40,41]. First, GATK SplitIntervals was used to define 2600 evenly sized genomic intervals over which to parallelise variant calling. HaplotypeCaller was then executed in parallel over these 2600 intervals, using mode -ERC GVCF to emit 2600 genomic variant call format (GVCF) files per sample. GATK GatherVcfs GATK v4.4.0.0 [41] was used to create one GVCF per sample. GATK GenomicsDBImport GATK v.4.4.0.0 [41] was used to joint genotype the 2600 intervals and finally gathered into one cohort VCF, which was then sorted and indexed by GATK SortVcf GATK v.4.4.0.0.

The candidate gene region was identified and plus 1000 bp of flanking sequence was used to extract variants which were subjected to variant annotation with snpEff v. 4.3t [42]. A custom ARS-UI_Ramb_v2.0 genome snpEff database was built with the snpEff ‘build’ tool and GTF downloaded from NCBI (https://www.ncbi.nlm.nih.gov/datasets/genome/GCF_016772045.1/, accessed on 17 October 2023). From the variants in the candidate region, variants present in the two affected sheep with a predicted functional effect of ‘low’, ‘high’ or ‘moderate’ were considered for further analysis.

#### 2.3.3. Validation of the NC_056057.1:g.107930611C>T Variant

In silico tools MutPred2 (http://mutpred.mutdb.org/, accessed on 1 November 2024) [43], PolyPhen2 (http://genetics.bwh.harvard.edu/pph2/, accessed on 1 November) [44], LIST-S2 (https://list-s2.msl.ubc.ca/, accessed on 1 November 2024) [45], and Meta SNP (https://snps.biofold.org/meta-snp/index.html, accessed on 1 November 2024) [46] (Meta SNP includes PANTHER [47], PhD-SNP [48], SIFT [49] and SNAP [50]) were used to assess pathogenicity of amino acid substitutions using the normal sheep CLCN1 protein (XP_004008185.4) and the predicted amino acid change as input [51].

A custom TaqMan assay (Applied Biosystems, Carlsbad, CA, USA) was developed to genotype the likely causal SNP in the *CLCN1* gene. The qPCR was performed on a ViiA 7, StepOne or 7500 system (Applied Biosystems, USA) in a final reaction volume of 12.5 µL. Reaction mix consisted of 1x TaqMan Genotyping Master Mix (Applied Biosystems, USA), 900 nmol/L of allele specific primers 5′-GGGAGTCGGCTGCTGTTT-3′ and 5′-GAACAGGCAAGTGCACTGATG-3′, 250 nmol/L of allele specific probes 5′-VIC-CCTCCAAGAGGTGTCCCA-NFQ-3′ (wildtype) and 5′-FAM-CCTCCAAGAGATGTCCCA-NFQ-3′ (mutant) and 5–50 ng of genomic DNA. Cycling parameters were pre-read hold at 60 °C for 30 s, initial denaturation at 95 °C for 10 min, followed by 45 cycles of denaturation at 95 °C for 15 s, annealing and extension at 60 °C for 60 s and a final post-read stage at 60 °C for 30 s.

## 3. Results

### 3.1. Characterisation of the Disease Phenotype

#### 3.1.1. History and Clinical Examination

Lambs examined were alert and normal between episodes and were in good body condition. On disturbing the lambs, eight of the thirteen fell to lateral recumbency and exhibited tetanic spasms but were alert. The remaining five lambs showed a stiff, rigid hind limb gait and moved away hopping on their hindlimbs. Recumbent lambs rapidly recovered and initially had a stiff gait while walking away. Lambs appeared to completely recover within five to 30 s and some had resumed eating. Three lambs examined following episodes of collapse had a palpable tachycardia, normal menace response and a slightly elevated body temperature (40.3 °C). At five months of age, of the remaining nine affected lambs, only eight showed the characteristic clinical presentation of episodic collapse and muscle rigidity induced by movement (Video S1).

Given the number of affected animals, the possibility of mating related animals, and the lack of breeding stock displaying clinical signs, the mode of inheritance was suspected to be recessive.

#### 3.1.2. Clinical Pathology

Biochemistry and haematology results for the four lambs are included in Supplementary Table S2. Creatine kinase (CK) was mildly elevated in two of four lambs, with CK ranging from 238 to 357 U/L. Aspartate aminotransferase (AST) was mildly elevated in three of seven lambs, with values ranging from 115 to 179 U/L. Globulins were mild to moderately decreased in all lambs. Two lambs had mild elevations in gamma-glutamyl transferase (GGT). All four lambs had mildly elevated urea. There were no abnormalities in calcium, magnesium, potassium or glutathione peroxidase. Haematology was relatively unremarkable apart from all lambs having mildly reduced mean cell haemoglobin content (MCHC); other changes were marginal.

#### 3.1.3. Electromyography

Electromyography of affected animals under field conditions showed spontaneous bursts of electrical activity of increased frequency and amplitude compared to the background baseline (Supplementary Figure S1). The electromyography technique did not allow for connection to a speaker and evaluation of audio.

#### 3.1.4. Gross Pathology and Histopathology

There were no gross abnormalities in the post mortem of four affected sheep. In H&E-stained skeletal muscle sections, there was mild variation in myofibre size and occasional myofibres were hypereosinophilic, mildly round and swollen or shrunken (Supplementary Figure S2). Multifocally, in muscle sections of two of the four affected sheep, there was mild, lymphoplasmacytic and eosinophilic myositis.

In one affected animal, the space between sciatic nerve fascicles and the surrounding perineurium was multifocally filled with bright eosinophilic, homogenous fluid, interpreted as proteinaceous fluid or oedema. Within the radial nerve of the same sheep, along the epineurium, there were multifocal, mild infiltrates of eosinophils and occasional macrophages, lymphocytes and plasma cells, interpreted as mild, multifocal, eosinophilic perineuritis.

For all four affected sheep, there were no significant histologic abnormalities in sections of liver, lung, spleen, kidney, brain or cervical, thoracic and lumbar spinal cord. LFB staining of sciatic nerve for sheep 1 and 2, and radial nerve from sheep 1, showed normal myelination.

#### 3.1.5. Parentage Verification

Of the 31 sampled, 2 rams qualified as the sire for 9 of the affected lambs. The remaining 4 affected lambs were presumed to be sired by another ram that was no longer available for testing, given a biological relationship was excluded with each of the 31 sires.

### 3.2. Candidate Genes

Searching the OMIA and OMIM databases for disease with similar clinical and pathological presentation identified *CLCN1* as a strong candidate gene. An OMIM search for “myotonia and channelopathy” yielded 12 results—8 conditions and 4 genes (Supplementary Table S3).

Review of OMIA revealed 16 disorders across non-laboratory domestic species that had clinical similarities to myotonia congenita (Table 1 and Table S4); however, many could be excluded based on concurrent clinical signs, clinical pathology, advanced clinical diagnostics and/or histopathology. Non-dystrophic hereditary myotonia may also occur with variants in *SCN4A*, including paramyotonia congenita, hyperkalaemic periodic paralysis (HYPP) and sodium channel myopathies or potassium aggravated myotonia in humans [4,5,26], and HYPP in horses. In HYPP, muscle stiffness typically worsens with exercise [4,5,26], which was not observed in this case. HYPP in horses can be associated with collapse in severe cases, episodic muscle tremors, weakness and paralysis, with increased potassium levels during episodes [52,53], not considered to be consistent with the clinical presentation in affected Merino sheep.

Myotonia congenita was considered the likely diagnosis based on clinical presentation and absence of dystrophic muscle changes histologically. Myotonia congenita has been reported in eight non-laboratory animal species, with a variant in *CLCN1* confirmed in seven species (Table 1 and Table S4). The only other reported case of ovine myotonia segregated with a variant in *CLCN1* [15].

### 3.3. Identification and Validation of a Likely Causal Variant in CLCN1

#### 3.3.1. Genome Wide Association Study (GWAS)

**Figure 1:**
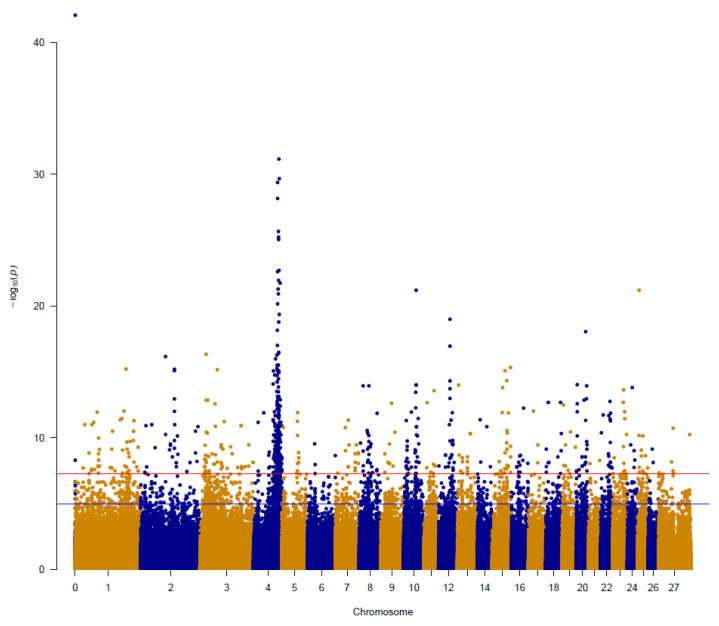
Manhattan plot of a basic case/control GWAS analysis associated with myotonia phenotype. Alternating yellow and blue dots indicate different chromosomes. The *p*-values obtained from GWAS are presented on −log10 scale. The blue line represents a suggestive significance threshold (−log10(*p*) = 5) and the red line represents the genome-wide significance threshold (−log10(*p*) = 8). The peak on ovine chromosome 4 overlaps with the position of the functional candidate gene CLCN1.

A basic case/control genome wide association analysis identified a significant peak associated with the myotonia phenotype on ovine chromosome 4 (Figure 1) (Supplementary Table S5). The peak consisted of 107 SNP, with the most significant SNP (OAR4_114218496.1) on chromosome 4 lying adjacent to the functional candidate gene *CLCN1*. The GWAS results therefore support *CLCN1* as a functional and positional candidate gene. Other genes associated with myotonia-like diseases such as *SNC4A*, *ATP2A1, KCNG1*, and *SLC7A10* are located on chromosome 11, 24, 13 and 14, respectively, and were therefore not further investigated.

#### 3.3.2. Whole Genome Sequencing Analysis

Whole genome sequencing of 2 affected lambs and 23 unrelated control animals identified 525 variants, in the *CLCN1* gene region (Supplementary Table S6), including 84 and 85 variants that were present in the two affected lambs. Of the 525 variants identified across all 25 animals, 13, 5 and 0 were predicted to have a low, moderate or high impact on protein function, respectively. Of these, only 4 were present in the two affected sheep: 3 variants with a low predicted impact (2 synonymous variants and an intronic variant in close proximity to a splice site) that were also present in several of the control sheep, and 1 missense variant with a predicted moderate impact that was also the only variant in the whole dataset that was only present in the two affected sheep and not present in any of the 23 control sheep. This missense variant in *CLCN1* (NC_056057.1:g.107930611C>T, XM_004008136.5:c.844C>T) was considered as the only likely causal variant in the *CLCN1* region.

#### 3.3.3. Validation of the NC_056057.1:g.107930611C>T Variant

The NC_056057.1:g.107930611C>T variant is predicted to replace a proline with a serine residue (XP_004008185.4:p.(P282S)). This amino acid change was consistently predicted to be deleterious (Table 2) using eight in silico tools recommended to assess pathogenicity of missense variants in animals [125]. Alignment of CLCN1 protein sequence by MUSCLE [126] across multiple species demonstrates that this is a highly conserved residue (Supplementary Figure S3). The *CLCN1* missense variant was therefore identified as being likely causal for these affected animals and has been assigned in OMIA the identifier omia.variant:1664.

The NC_056057.1:g.107930611C>T variant had been previously identified as an ovine SNP as part of the International Sheep Genomics Consortium’s (ISGC) 1000 genomes project [127] and had been assigned with an European Variation Archive identifier rs3487175777 (https://www.ebi.ac.uk/eva/?variant&accessionID=rs3487175777, accessed on 31 October 2024). To assess allele frequency for the variant in the 1000 genomes project, we obtained the vcf of chromosome 4 from the CSIRO data access portal [127] and metadata from Figshare [128]. The variant was found in heterozygous form in 3 out of 935 animals, making the allele frequency 0.16% in that cohort of animals.

The SNP genotyping assay showed that 12 of the 13 owner-reported affected sheep were homozygous for the likely causal variant and the 2 rams that were identified to have sired affected lambs were heterozygous for the variant. One additional ram being used in the flock was also heterozygous, while the remaining rams and eighteen Merino control animals from an unrelated flock were homozygous wildtype.

## 4. Discussion

Myotonia congenita is a skeletal muscle channelopathy reported to occur naturally in humans as well as mice, water buffalo, sheep, goats, dogs, cats, horses, pigs and cattle, for which associated variants in *CLCN1* have been reported in each species except cattle which remains undetermined [4,10,11,12,13,14,15,16,29]. A thorough clinical and pathologic investigation supported a diagnosis of myotonia congenita in Merino sheep, allowing identification of a functional candidate gene and the identification and validation of the likely causal variant in *CLCN1*. This is the first report of myotonia congenita in Merino sheep, with previous reports in Rasa Aragonesa and an unspecified breed of sheep in the USA [15,29].

### 4.1. Characterisation of the Disease Phenotype

#### 4.1.1. Clinical Examination

Previously reported clinical findings in myotonia congenita in animals are summarised in Supplementary Table S4. Clinical presentation can vary between and within species. Affected animals often have episodes of muscle stiffness and rigidity with variable collapse, associated with initiation of movement or a sudden visual or auditory stimulus (startle response), and improvement of clinical signs with exercise (warm-up phenomenon) [13,15]. Episodes are of short duration and vary from seconds to minutes in case reports. Muscle hypertrophy is often described but can be variable in severity, and may be generalised [13] or more pronounced in particular muscle groups, such as proximal or distal limbs [2,14,17,129], epaxial muscles [14,17], and tongue [2,17]. Muscle percussion may also stimulate a classic myotonic dimple, which is a localised sustained muscle contraction [14]. Clinical signs may be worse in cold weather [28]. Neurologic examination is usually unremarkable [2]; however, occasionally, there may be reduced reflexes due to muscle stiffness or delayed relaxation of muscles [17,57,64] or in cats, species related differences [17]. While myotonia congenita is reported in a number of dog breeds, the Miniature Schnauzer appears to have unique dental and facial abnormalities associated with the condition [67,68].

Similar to previous cases of ovine myotonia congenita, Merino sheep demonstrated the classic clinical presentation of falling over with muscle rigidity, often induced by movement or a sudden response [15,29]. Episodes were transient and sheep quickly recovered and were clinically unaffected between episodes. The first clinical sign in a previous investigation in sheep was dirty fleeces due to lambs falling over [29]. Affected Merino sheep in the current investigation did not have obvious muscle hypertrophy, in contrast to previous cases of ovine myotonia congenita, for which heavier muscling [29] and muscular enlargement particularly of the hindlimb [15] were reported. While lambs are likely to develop clinical signs from a young age, identification of affected lambs may not occur until yarding and handling for weaning in extensive production systems.

Summaries of clinical signs of myotonia congenita in humans can be found on OMIM for autosomal dominant (OMIM:160800) and autosomal recessive forms (OMIM:255700) [31] (Supplementary Table S3). There are some similarities to clinical signs reported in animals, including delayed muscle relaxation after contraction, muscle hypertrophy, dysphagia and muscle stiffness or weakness, often more pronounced in extremities [31]. Muscle pain is sometimes described in humans [6,31]. While animals are generally not noted to have muscle pain, this may be because mild pain in animals can be difficult to assess.

#### 4.1.2. Clinical Pathology

There are typically no significant haematologic abnormalities in myotonia congenita. Complete blood counts have been reported as unremarkable in dogs [25,66,72] or within limits [2,22] or with non-specific and inconsistent findings in cats [57]. The reduced MCHC in all lambs in the current study may be secondary to artefactual swelling, with an approximate 24 to 48 h delay between sample collection and analysis.

Biochemistry in cases of myotonia congenita may be used to exclude other differential diagnoses. Animals with myotonia congenita often have the muscle enzyme CK either within reference range [2,17,22,23,57,66] or mildly elevated [2,17,18,24], occasionally more substantially elevated [59]. Similarly in humans, CK may be normal or mildly elevated [26]. Biochemistry is otherwise typically unremarkable [2,18,29] or changes are described as secondary, such as due to stress [57]. No clinical pathology was reported in previous cases of myotonia in water buffalo, pigs and the single case of a Friesland calf [13,14,20].

Clinical pathology in previous cases of ovine myotonia has been limited to two affected animals, in which there was normal complete blood counts, biochemistry including CK, and urinalysis [29]. While a mild elevation in CK was detected in two of four Merino lambs in the current study, these mild elevations can be commonly seen with handling or could be secondary to episodes of falling.

The hypoglobulinaemia in all lambs may be age related, with the provided laboratory reference range for adults. Episodes of collapse may have hindered normal suckling, however a comparison of globulins in clinically normal lambs in the flock is lacking. The elevated urea is most likely pre-renal due to dehydration associated with yarding and reduced water intake, although a urine specific gravity would be required to confirm this. There were no significant histologic abnormalities within sections of kidney to indicate a likely renal cause. Normal calcium and magnesium levels exclude hypocalcaemia or hypomagnesaemia as causes of weakness and collapse.

#### 4.1.3. Electromyography

Electromyography provides one of the key diagnostic modalities for investigating myotonia congenita, when interpreted in context of typical clinical signs and lack of significant necrosis or inflammation in muscle biopsies. Electromyography findings in cases of myotonia congenita are characterised by waxing and waning discharges of high frequency and amplitude, with positive or negative waves [29,57]. When connected to audio, the discharges sound like a diving aeroplane (‘dive bomber’) or an accelerating and decelerating motorcycle [2,8,14,18,57]. In two feline reports, discharges were described as sounding like a swarm of bees [17,22]. These discharges can be spontaneous or may also be stimulated with needle insertion, movement, or with muscle percussion [2,28,57]. Heating and cooling muscle shortens and lengthens duration of myotonic discharges, respectively [60,70]. Myotonic discharges reduce in duration with repeated percussion [60].

Myotonic discharges on electromyography are not a pathognomonic finding of myotonia congenita and may occur with other forms of myotonia [130]. Muscle hypertrophy and stiffness with spontaneous high frequency waxing and waning discharges on electromyography have been reported with myotonia associated with hyperadrenocorticism in dogs [131]. Additionally, high-frequency discharges or complex repetitive discharges can be seen on electromyography of humans and animals with a number of other myopathic and neuropathic diseases [130,132,133]. Electromyography findings should therefore be interpreted in context of clinical presentation and histopathology.

Previous electromyography on sheep with myotonia congenita described waves up to 1 mV ranging from 50 to 100 impulses/second [29]. A modified electromyography technique was adapted in this study as sheep were located on a remote property in NSW and were unable to be transported the considerable distance to the specialist veterinary clinic. In other animal species, electromyography is often conducted in heavily sedated or anaesthetised animals to avoid voluntary movements. However, this diagnostic procedure is routinely conducted in humans without sedation or anaesthesia and the non-sedated sheep in this study were calm and recumbent when examined, and any sudden movements were recorded alongside the electromyography for interpretation. While spontaneous bursts of electrical activity of increased frequency and amplitude compared to the background baseline were observed in affected sheep, these findings should be considered with caution, considering the limitations in this field study.

Evaluation of clinical signs prior to and after administration of a neuromuscular junction blockade has assisted in previous investigations, particular prior to identification of causal variants. In myotonia congenita, there is persistence of electromyography abnormalities and abnormalities such as the myotonic dimple, after administration of a neuromuscular junction blockade [60]. Conversely, electromyography myotonic discharges did not occur after intramuscular injection of lignocaine in a dog [60].

When tested, nerve conduction velocity is typically normal in cases of confirmed or suspected myotonia congenita [2,60,70]. On repetitive nerve stimulation testing, a decrement has been reported in cats [17] and Chow Chow dogs with myotonia congenita.

#### 4.1.4. Gross Pathology and Histopathology

Muscle biopsy in myotonia congenita may show variation in myofibre size, myofibre hypertrophy, or may be unremarkable. In the two previous reports of myotonia congenita in sheep, pathology changes have been limited to grossly described muscular enlargement most significant within the hind part [15] and, histologically, hypertrophy of type I and type II myofibres in the biceps femoris [29]. Muscle biopsies in pigs and water buffalo showed occasional hypertrophic muscle fibres but were otherwise unremarkable [13,14]. Moderate hypertrophy of muscle fibres has been described in goats, with occasional degenerative changes and central nuclei [134]. Previous cases of myotonia congenita in cats have described mild to moderate muscle fibre hypertrophy with one case of more marked hypertrophy, with variable reports of occasional central nuclei and myofibre degeneration with proliferation of sarcolemmal nuclei [2,17,23,57]. In one feline case, there were clear sarcoplasmic vacuoles [2]. Reports in dogs vary from mild hypertrophic myofibres [18,58,61,64], moderate variability in myofibre size [59] to marked variation in myofibre size [60], in the absence of significant concurrent muscle pathology. There may be occasional concurrent minor findings such as central nuclei or increased interfibrillar fat droplets [18]. Typically, there is no change in fibre type distribution [17,18,24,60,64], with some exceptions [72].

Confirming histopathologic changes are absent, or limited to hypertrophy with mild changes, can assist in differentiation from other myopathies. Dystrophic myopathies, such as dystrophin-deficient myopathies, result in polyphasic muscle degeneration and regeneration, variation in myofibre diameter and dystrophic calcification [2,28]. Glycogen storage diseases involving muscle, such as glycogen storage disease type IV in Norwegian Forest cats, may have muscle degeneration and atrophy, intracytoplasmic PAS-positive inclusions and involvement of other body systems [28]. Histopathology could also assist in differentiation acquired or immune-mediated myopathies, such as polymyositis.

The lack of significant histologic changes in the Merino sheep was consistent with the suspected diagnosis of myotonia congenita. The multifocal lymphoplasmacytic and eosinophilic myositis was not a consistent finding (present in two of four lambs) and was mild. Anecdotally, mild lymphoplasmacytic myositis, with or without eosinophils, is not uncommonly observed in sheep post mortems, postulated by the authors to possibly be associated with *Sarcocystis* sp. Infection, which can be frequently observed in muscle of sheep [135].

The Merino sheep in the current study had no consistent significant gross or histologic muscle hypertrophy. Toll et al. [2] implemented imaging software to quantify an increased mean myofibre area compared to muscle from an aged-matched control cat. This could be considered to further assess for potentially mild hypertrophy in these sheep should breed- and age-matched control muscle become available.

Frozen muscle sections allow a broader array of special stains and biochemical analysis compared to formalin-fixed specimens when investigating suspected myopathies. However, no abnormalities in mitochondrial distribution and enzyme reactivities have been found within canine cases in which this has been evaluated [18]. In the present study, frozen muscle samples for histochemical staining were unable to be taken in the field investigation.

Advanced histochemistry and transmission electron microscopy (EM) have been performed on selected investigations of myotonia congenita. EM in two cats with myotonia congenita found mild dilation of transverse (T) tubules [2], while in dogs found normal myofibres [18]. Goats with myotonia congenita were found to have PAS positive diastase resistant material in a reticulated form within muscle, as well as increased calcium within muscle, confirmed with alizarin red S staining and biochemical analysis [134]. EM on affected goats showed multifocal t-tubule changes including increased density and electron-dense material within t-tubules, proliferation of tubular elements, dilatation of parts of the sarcoplasmic reticulum and mitochondrial abnormalities [134]. Further sarcolemmal changes were observed on EM stains, and the EM changes were interpreted to be likely associated with myotonia [134]. Indirect immunofluorescence used in an investigation of feline myotonia congenita determined the underlying abnormality was likely due to ion channel dysfunction instead of abnormal location, with similar chloride channel staining in affected and control cats [17].

### 4.2. Identification and Validation of a Likely Causal Variant in CLCN1

The combination of a very detailed clinical and histopathologic investigation led to the diagnosis of myotonia congenita. Myotonia congenita in humans and animals is caused by variants in the *CLCN1* gene and information about genetics, including known causal variants in animals, are summarised in Table 1 and Table S4. We therefore investigated this functional candidate gene for likely causal variants in two affected Merino sheep using whole genome sequencing data, identified a missense variant (NC_056057.1:g.107930611C>T, XM_004008136.5:c.844C>T; XP_004008185.4:p.(P282S)), and confirmed *CLCN1* as positional candidate gene in a GWAS analysis. The identified variant is different to the previously reported missense variant (XP_004008185.4:p.(E93K)) in Rasa Araginosa sheep [15].

In genetic investigations, it can be difficult to prove causality of a suspected variant, and human recommendations were therefore followed to assess likely pathogenicity in this case. Using the standards for the interpretation of human sequence variants (American College of Medical Genetics (ACMG) criteria [136]), the identified variant (NC_056057.1:g.107930611C>T, XM_004008136.5:c.844C>T; XP_004008185.4:p.(P282S)) fulfills multiple criteria of a pathogenic variant (Table 3).

The variant did not fulfill criteria that would support a benign ACMG classification, except for the fact that one of the owner-reported affected sheep was homozygous normal for the identified variant in the SNP genotyping assay (ACMG criteria BS4: Lack of segregation in affected members of a family). This sheep was unfortunately not available for a follow-up investigation after DNA testing for the variant, and was not one of the animals from which blood or tissue samples were collected for clinical pathology or histopathology. Video evidence of the phenotype collected during the first farm visit did not allow identification of individual sheep in the group, but it was noted that not all sheep collapsed and that some of the owner reported affected sheep just presented with a stiff hind limb gait and ataxia. This sheep was noted at the time of the electromyography visit at five months of age to not present with the typical clinical presentation. It is therefore possible that this sheep may have been suffering from a non-genetic form of lameness or ataxia during the first farm visit.

Wider screening of sheep with the developed SNP genotyping assay will allow better understanding of how widespread this variant is and assist farmers in making informed decisions regarding animal selection. The allele frequency of this variant in the wider Merino population should be considered in future management decisions. As for other rare recessive single-gene diseases with relatively low to moderate impact on animal welfare, testing for this variant should be considered in flocks with suspected or confirmed cases of myotonia congenita. The variant could also be added to SNP panels that are used by sheep breeders for the genetic evaluation of their animals to identify at risk flocks. Based on the understanding that this variant is consistently predicted to have a deleterious impact on the *CLCN1* protein and is likely disease causing, any breeding of two animals that both carry at least one copy of the variant should be avoided to reduce the risk of affected animals been born. This can be easily achieved in commercial sheep flocks by ensuring that all rams are homozygous for the normal allele. In the current case, rams were screened for the myotonia variant to allow informed breeding decisions and to avoid at-risk matings.

## 5. Conclusions

Myotonia congenita is recognised as a hereditary myopathy in a number of veterinary species and should be considered a differential diagnosis for short, periodic episodes of muscle rigidity and/or collapse. Diagnosis of myotonia congenita in animals relies on clinical examination, electromyography and muscle biopsy, and when available, genetic testing to confirm the causative variant. Variants in *CLCN1* have been identified in human, mice and eight companion and production animal species. *CLCN1* should therefore be considered a candidate gene for investigating suspected myotonia congenita if identified in new species or breeds. Identifying a causative variant in Merino sheep in this study allowed the development of an SNP genotyping assay, identification of carrier animals, and modification of the breeding program to reduce the likelihood of breeding further affected animals. The availability of a diagnostic test will allow efficient screening of additional suspected affected or carrier Merino sheep.

## Figures and Tables

**Table 1:** Myotonia congenita associated with *CLCN1* variants in animals with differential diagnoses based on clinical presentation, as listed in Online Mendelian Inheritance in Animals (OMIA) [27]. Detailed information is provided in Supplementary Table S4.

OMIA ID	Name of Condition	Gene	Species	Breed	Mode of Inheritance
000698-9925	Myotonia congenita	*CLCN1*	Goat	Inbred line [8,16] Goats from Tennessee, USA [54,55]	AD
000698-9940	Myotonia congenita	*CLCN1*	Sheep	Rasa Aragonesa [15] VBO:0015863	AR
		ND	Sheep	Unspecified [29]	AR
		*CLCN1*	Sheep	Merino; VBO:0014151	AR
000698-9462	Myotonia congenita	*CLCN1*	Water Buffalo	Murrah buffalo [13] VBO:0000068	AR
000698-9823	Myotonia congenita	*CLCN1*	Pig	Unspecified [14]	AR
000698-9796	Myotonia congenita	*CLCN1*	Horse	New Forest [21] VBO:0001026	AR
	Myotonia congenita (possible)	ND	Horse	Thoroughbred [56] VBO:0001083	ND
000698-9685	Myotonia congenita	*CLCN1*	Cat	Domestic Longhair [2,22] VBO:0100118	ND
		ND	Cat	Domestic Shorthair [2,57] VBO:0100119	ND
		*CLCN1*	Cat	Feral random bred [17]	AR
		*CLCN1*	Cat	Mixed breed [23]	ND
000698-9913	Myotonia congenita	ND	Cattle	Friesland [20]	ND
000698-9913	Myotonia congenita	*CLCN1*	Dog	American Bulldog [58] VBO:02000034	AR
		*CLCN1*	Dog	Australian Cattle Dog [59] VBO:02000088	AR
		ND	Dog	Chow Chow [60,61,62,63] VBO:0200361	ND
		ND	Dog	Cocker Spaniel [64] VBO:0200372	ND
		*CLCN1*	Dog	French Bulldog [24] VBO:0201455	ND
		ND	Dog	Great Dane [65] VBO:0200623	ND
		*CLCN1*	Dog	Jack Russell Terrier [66] VBO:0200724	ND
		*CLCN1*	Dog	Labrador Retriever [18] VBO:0200800	ND
		*CLCN1*	Dog	Miniature Schnauzer [19,67,68,69,70] VBO:0200896	AR
		*CLCN1*	Dog	Mixed breed dog [25]	ND
		ND	Dog	Miniature poodle [71] VBO:0201051	ND
		ND	Dog	Staffordshire Bull Terrier [72] VBO:0201296	ND
001723-9940	Ataxia, familial episodic spinocerebellar	*FGF14*	Sheep	Romney Marsh [73,74] VBO:0001582	AD with variable expressivity
001450-9913	Congenital muscular dystonia 1 (CMD1)	*ATP2A1*	Cattle	Belgian Blue [75,76] VBO:0000139	ND
001451-9913	Congenital muscular dystonia 2 (CMD2)	*SLCA5*	Cattle	Belgian Blue [76,77] VBO:0000139	AR
002084-9615	Dyskinesia, paroxysmal, PIGN-related	*PIGN*	Dog	Soft-Coated Wheaten Terrier [78] VBO:0201260	AR
002322-9615	Dyskinesia, paroxysmal, SOD1-related	*SOD1*	Dog	Markiesje dogs [79] VBO:0008069	AR
001543-9615	Dyskinesia, paroxysmal	Unknown	Dog	Bichon Frise [80] VBO:0200163	ND
			Dog	Border Terrier [81,82] VBO:0200194	ND
			Dog	Chinook [83] VBO:0200353	AR
			Dog	Doberman Pinscher [84] VBO:0200442	ND
			Dog	English Bulldog [85] VBO:0200485	ND
			Dog	German Spitz [86] VBO:0200585	ND
			Dog	Jack Russell Terrier VBO:0200724 and Labrador Retriever VBO:0200800 [87]	ND
			Dog	Norwich Terrier [88,89] VBO:0200962	ND, may be multigenic or complex
			Dog	Pomeranian VBO:0201043, mixed breed, poodle VBO:0201048 [90]	ND
			Dog	Scottish Terrier [91,92]	Possible AR
			Dog	Various breeds, most commonly poodle [93]	ND
			Dog	Welsh Terrier [94] VBO:0201412	ND
001543-9685	Dyskinesia, paroxysmal	Unknown	Cat	Sphynx [95] VBO:0100230	ND
001466-9615	Exercise-induced collapse	*DNM1*	Dog	Labrador Retriever VBO:0200800 [96,97]	AR
-	Exercise-induced collapse	Unknown	Dog	Border collie VBO:0200193 and related breeds [98,99,100]	Complex polygenetic trait
001592-9615	Episodic falling over, BCAN related	*BCAN*	Dog	Cavalier King Charles Spaniel [101,102] VBO:0200309	AR
000689-9796	Hyperekplexia, GLRA1-related	*GLRA1*	Horse	Peruvian Paso [103] VBO:0001042	ND
000689-9615	Hyperekplexia, GLRA1-related	*GLRA1*	Dog	Miniature Australian Shepherd Dogs [104] VBO:0200881	AR
000689-9913	Hyperekplexia, GLRA1-related	*GLRA1*	Cattle	Polled Hereford [105,106,107,108] VBO:0000341	AR
001594-9615	Hyperekplexia (Startle disease), SLC6A5-related	*SLC6A5*	Dog	Irish Wolfhound [109] VBO:0200706	AR
				Spanish Greyhound [110] VBO:0201274	AR
000785-9796	Hyperkalemic Periodic Paralysis (HYPP)	*SCN4A*	Horse	Quarter horse [53,111,112,113,114] VBO:0001057	Autosomal incomplete dominant
002483-9913	Neuromuscular channelopathy, KCNG1-related	*KCNG1*	Cattle	Bovine (Belgian Blue x Holstein crossbred calf) [115]	Unknown, possible de novo variant
002645-9615	Paradoxical pseudomyotonia;	*SLC7A10*	Dog	English Cocker Spaniel VBO:0200486 and English Springer Spaniels VBO:0200497 [116,117]	AR
001464-9913	Pseudomyotonia, congenital	*ATP2A1*	Cattle	Chianina VBO:0000178 (variant later reported in other breeds Marchigiana VBO:0000291, Romagnola) VBO:0000360 [118,119,120]	AR
		*ATP2A1*	Cattle	Romagnola [121] VBO:0000360	AR
		*ATP2A1*	Cattle	Dutch Improved Red and White cross-breed calf [122]	AR
000929-9940	Spastic syndrome	ND	Sheep	Bluefaced Leicester [123] VBO:0001344	ND
000929-9913	Spastic syndrome	ND	Cattle	Numerous breeds (Holstein-Friesian, Guernsey, Ayrshire, Brahma cross, Shorthorn, Poll Hereford) [124]	ND

ND = not determined; AR = Autosomal recessive; AD = Autosomal dominant.

**Table 2:** Prediction of pathogenicity of XP_004008185.4:p.(P282S) using MutPred2 (http://mutpred.mutdb.org/, accessed on 1 November 2024), PolyPhen2 (http://genetics.bwh.harvard.edu/pph2/, accessed on 1 November 2024), LIST-S2 (https://list-s2.msl.ubc.ca/, accessed on 1 November 2024), Meta SNP, PANTHER, PhD-SNP, SIFT and SNAP (https://snps.biofold.org/meta-snp/index.html), accessed on 1 November 2024.

In Silico Tool	Analysis	Result	Score	Comment
MutPred2	MutPred2	Altered transmembrane protein, altered metal binding, loss of catalytic site at F279	0.806	>0.50 suggests pathogenicity
PolyPhen2	Hum Div	Probably damaging	1.000	>0.15 to 0.85 possibly damaging; >0.85 probably damaging
	Hum Var	Probably damaging	0.995	>0.15 to 0.85 possibly damaging; >0.85 probably damaging
LIST-S2	LIST-S2	Deleterious	0.9915	>0.85 variant is considered deleterious
MetaSNP	PANTHER	Disease	0.975	>0.5 variant is predicted ‘Disease’
	PhD-SNP	Disease	0.929	>0.5 variant is predicted ‘Disease’
	SIFT	Disease	0	<0.05 variant is predicted ‘Disease’
	SNAP	Disease	0.735	>0.5 variant is predicted ‘Disease’
	Meta-SNP	Disease	0.832	>0.5 variant is predicted ‘Disease’

**Table 3:** American College of Medical Genetic criteria [136] supporting pathogenicity of the XM_004008136.5:c.844C>T; XP_004008185.4:p.(P282S) variant.

ACMG Code *	ACMG Criteria	Support for XP_004008185.4:p.(P282S)
PS4	The prevalence in affected individuals is significantly increased in affected animals compared to controls	12 of 13 owner reported affected animals were homozygous for the variant, 18 Merino controls from an unrelated flock were homozygous wildtype
PM1	Located in a mutational hot spot/or critical and well-established functional domain without benign variation	p.(P282S) is located in an intramembrane helical domain ranging from amino acids 281–290 (https://www.uniprot.org/uniprotkb/P35523/feature-viewer, accessed 31 October 2024) Variants in this domain at position 285, 286 and 290 are reported to result in loss of/or reduced chloride transport and are classified as pathogenic (ClinVar, https://www.ncbi.nlm.nih.gov/clinvar/, accessed 31 October 2024) Human variant (p.(P282R)) is considered to impact spatial conformation of this domain due to the rigidity of the proline residue [137] MutPred predicts that ovine p.(P282S) alters the transmembrane protein, alters metal binding and results in the loss of a catalytic site at F279 (Table 2)
PM2	Absent from controls (or at extremely low frequency if recessive) in 1000 genomes project of similar	Allele frequency in ISGC 1000 genomes project [127] is very low (0.16%: 3 out of 935 sheep were heterozygous), condition is recessive and not lethal
PM5	Novel missense change at an amino acid residue where a different missense change determined to be pathogenic has been seen before	A missense change p.(P282R) was reported in a human patient with myotonia congenita [137]
PP1	Co-segregation with disease in multiple affected family members in a gene definitively known to cause the disease	The variant co-segregated with 12 affected sheep and all confirmed sires (*n* = 2)
PP2	Missense variant in a gene that has a low rate of benign missense variation and where missense variants are a common mechanism of disease	ClinVar (https://www.ncbi.nlm.nih.gov/clinvar/, accessed 31 October 2024) reports 625 missense variants in human *CLCN1*—140 pathogenic or likely pathogenic, 25 likely benign or benign, 504 uncertain and 90 conflicting, information in animals is lacking information about confirmed benign variants but OMIA lists 5 missense variants in *CLCN1* as likely causal variants in animals (Supplementary Table S4)
PP3	Multiple lines of effect on the gene or gene product computational evidence support a deleterious	MutPred2, PolyPhen2, LIST-S2, PANTHER, PhD-SNP, SIFT, SNAP, Meta-SNP predict deleterious impact (Table 2)
PP4	Patient’s phenotype of family history is highly specific for a disease with a single genetic aetiology	Phenotype of affected sheep is specific for myotonia congenita in humans and multiple animal species

* PS/PM/PP = strong/moderate/supporting evidence for pathogenicity, respectively.

## Data Availability

The original contributions presented in this study are included in the article and the Supplementary Materials. The likely causal variant is listed in the European Variation Archive (rs3487175777) and in Online Mendelian Inheritance in Animals (omia.variant:1664) [27].

## Supplementary Materials

The following supporting information can be downloaded at: https://www.mdpi.com/article/10.3390/ani14243703/s1, Table S1: Overview of tests performed for each sheep. Table S2: Biochemistry and haematology of Merino sheep with myotonia congenita. Table S3: Human genes and conditions identified in a search of Online Mendelian Inheritance in Man (OMIM) for “myotonia” and “channelopathy”. Table S4: Detailed information about myotonia congenita in domesticated animals and other inherited diseases with similar presentation. A comprehensive review is provided for myotonia congenita and briefer summaries are provided for differential diagnoses. Table S5: Most significant SNP (−log10(*p*) = 8) identified in a PLINK case/control GWAS analysis associated with the myotonia phenotype. The functional candidate gene *CLCN1* is located on chromosome 4 (NC_019461.1:106,132,787–106,176,971). Table S6: Variants identified in whole genome sequencing data of 2 affected lambs (Y3190 and Y3404) and 23 unrelated control sheep in VCF format based on the ARS-UI_Ramb_v2.0 reference genome. The likely causal variant is highlighted in yellow. Variants with a predicted low or moderate impact on protein function are indicated by a light blue or red fill of the ’INFO’ column, respectively. Variants that are homozygous for the reference allele are highlighted by a green fill in the columns for individual sheep. Figure S1: Electromyography of an affected sheep showing a spontaneous myotonic discharge in the M. extensor carpi radialis lasting 0.8 s. Figure S2: Histopathology of skeletal muscle from a sheep affected with myotonia congenita. There is mild variation in myofibre size, with occasional myofibres that are shrunken or rounded and swollen, haematoxylin and eosin, ×20. Figure S3: Partial CLCN1 protein sequences of 15 species aligned by MUSCLE [126]. Video S1: An affected sheep with episodic collapse and muscle rigidity induced by movement.
